# Characterization of a Novel Chromosome-Encoded AmpC β-Lactamase Gene, *bla*_PRC–1_, in an Isolate of a Newly Classified *Pseudomonas* Species, *Pseudomonas wenzhouensis* A20, From Animal Farm Sewage

**DOI:** 10.3389/fmicb.2021.732932

**Published:** 2021-12-17

**Authors:** Peiyao Zhang, Xu Dong, Kexin Zhou, Tingting Zhu, Jialei Liang, Weina Shi, Mengdi Gao, Chunlin Feng, Qiaoling Li, Xueya Zhang, Ping Ren, Junwan Lu, Xi Lin, Kewei Li, Mei Zhu, Qiyu Bao, Hailin Zhang

**Affiliations:** ^1^Key Laboratory of Medical Genetics of Zhejiang Province, Key Laboratory of Laboratory Medicine, School of Laboratory Medicine and Life Sciences, Ministry of Education, Wenzhou Medical University, Wenzhou, China; ^2^The Second Affiliated Hospital and Yuying Children’s Hospital, Wenzhou Medical University, Wenzhou, China; ^3^Institute of Biomedical Informatics, School of Laboratory Medicine and Life Sciences, Wenzhou Medical University, Wenzhou, China; ^4^Department of Clinical Laboratory, Zhejiang Hospital, Hangzhou, China

**Keywords:** PRC-1, β-lactamase, *Pseudomonas*, AmpC, kinetic analysis, resistance

## Abstract

In this work, we characterized a novel chromosome-encoded AmpC β-lactamase gene, *bla*_PRC–1_, in an isolate of a newly classified *Pseudomonas* species designated *Pseudomonas wenzhouensis* A20, which was isolated from sewage discharged from an animal farm in Wenzhou, China. Susceptibility testing, molecular cloning, and enzyme kinetic parameter analysis were performed to determine the function and enzymatic properties of the β-lactamase. Sequencing and comparative genomic analysis were conducted to clarify the phylogenetic relationship and genetic context of the *bla*_PRC–1_ gene. PRC-1 is a 379-amino acid AmpC β-lactamase with a molecular weight of 41.48 kDa and a predicted pI of 6.44, sharing the highest amino acid identity (57.7%) with the functionally characterized AmpC β-lactamase PDC-211 (ARX71249). *bla*_PRC–1_ confers resistance to many β-lactam antibiotics, including penicillins (penicillin G, amoxicillin, and amoxicillin-clavulanic acid) and cephalosporins (cefazolin, ceftriaxone, and cefotaxime). The kinetic properties of PRC-1 were compatible with those of a typical class C β-lactamase showing hydrolytic activities against β-lactam antibiotics, and the hydrolytic activity was strongly inhibited by avibactam. The genetic context of *bla*_PRC–1_ was relatively conserved, and no mobile genetic element was predicted in its surrounding region. Identification of a novel β-lactamase gene in an unusual environmental bacterium reveals that there might be numerous unknown resistance mechanisms in bacterial populations, which may pose potential risks to human health due to universal horizontal gene transfer between microorganisms. It is therefore of great value to carry out extensive research on the mechanism of antibiotic resistance.

## Introduction

The genus *Pseudomonas*, first described by Professor Mikula in 1894, is one of the most common bacteria in the world and has been found in various natural environments, human clinical specimens, and infected plants (Peix et al., 2009). *Pseudomonas* is a diverse and complex genus with the largest number of known species (Lalucat et al., 2020). At present, 396 species and 21 subspecies of *Pseudomonas* are included in the List of Prokaryotic Names with Standing in Nomenclature (Parte, 2014). The genus *Pseudomonas* mainly includes species that are pathogenic to animals and humans (*Pseudomonas aeruginosa*), insects (*Pseudomonas entomophila*), and plants (*Pseudomonas syringae*); species that are plant commensals (*Pseudomonas stutzeri* and *Pseudomonas fluorescens*); and species used in bioremediation (*Pseudomonas putida*) (De Smet et al., 2017). Extensively studied and economically important species are the human opportunistic pathogen *P. aeruginosa* and the plant pathogen *P. syringae* (Silby et al., 2011). *P. aeruginosa* is the most common cause of infection among non-fermenting gram-negative bacteria, which mainly affects patients with weakened immune function (Behzadi et al., 2021).

β-lactamase is an enzyme that can hydrolyze the β-lactam ring and inactivate antibiotics before binding to penicillin-binding proteins (Li et al., 2007). The AmpC enzyme is a type of β-lactamase mediated by chromosomes or plasmids in most Enterobacteriaceae and non-fermenting species, such as *P. aeruginosa*. AmpC belongs to Class C β-lactamase in the Ambler molecular structure classification and group I in the Bush-Jacoby-Medeiros functional classification of β-lactamases (Bush et al., 1995). AmpC β-lactamases are able to confer resistance to most penicillins and cephalosporins and cannot be inhibited by normal β-lactamase inhibitors, such as clavulanic acid and tazobactam (Bush et al., 1995; Livermore, 1995), but it can be strongly inhibited by avibactam (Docquier and Mangani, 2018). AmpC β-lactamases are usually not expressed or are underexpressed in *E. coli* because of a weak promoter and a transcriptional attenuator preceding the *ampC* gene; however, due to mutations in β-lactamases or induction by specific β-lactams, these enzymes can be expressed at high levels and consequently increase the minimum inhibitory concentrations (MICs) of β-lactams (Fisher and Mobashery, 2014; Juan et al., 2017). This effect is attributed to specific spontaneous mutations in the promoter or attenuation consensus sequence of the *ampC* gene (Haenni et al., 2014). It is worth noting that the diversity caused by mutations and the overexpression caused by antimicrobial induction of these enzymes are evolving to confer resistance to various β-lactam antibiotics. Therefore, the development of new antibiotics and novel antibacterial therapeutic strategies is urgently needed (Bonomo, 2017).

In this study, based on the complete genome sequencing of an isolate of a newly classified *Pseudomonas* species, *Pseudomonas wenzhouensis* A20, we characterized a novel chromosome-encoded AmpC β-lactamase, designated PRC-1, and analyzed the kinetic parameters of the β-lactamase. Discovering a novel resistance gene in an environmental bacterium provides valuable information for the treatment of infectious diseases.

## Materials and Methods

### Bacterial Strains

The strain *P. wenzhouensis* A20 carrying the novel β-lactamase gene *bla*_PRC–1_ was isolated from sewage discharged from an animal farm in Wenzhou, China. It was initially identified using a bioMérieux VITEK 2 Compact Instrument (BioMérieux, Marcy L’etoile, France). Further species identification was carried out by 16S ribosomal RNA (rRNA) gene sequencing and finally verified using average nucleotide identity (ANI) (Lachance et al., 2020). The bacterial strains and plasmids used in this work are listed in Table 1.

**TABLE 1:** Bacterial strains and plasmids used in this work.

Strain or plasmid	Characteristics	Source
**Strain**		
A20	The wild-type strain of *P. wenzhouensis* A20	This study
DH5α	*Escherichia coli* DH5α was used as a host for the cloning of the *bla*_PRC–1_ gene	Our laboratory collection
BL21	*Escherichia coli* BL21 was used as a host for the expression of PRC-1	Our laboratory collection
ATCC 25922	*Escherichia coli* ATCC 25922 was used as a quality control strain for antimicrobial susceptibility testing	Our laboratory collection
pUCP24-*bla*_PRC–1_/DH5α	DH5α carrying the pUCP24 vector with the *bla*_PRC–1_ gene with its upstream promoter region	This study
pCold I-*bla*_PRC–1_/BL21	BL21 carrying the pCold I vector with the open reading frame of the *bla*_PRC–1_ gene	This study
**Plasmid**		
pUCP24	Cloning vector for the PCR products of the *bla*_PRC–1_ gene with its upstream promoter region, GEN*^r^*	Our laboratory collection
pCold I	Expression vector for the PCR products of the ORF of the *bla*_PRC–1_ gene, AMP*^r^*	Our laboratory collection

*^r^ Resistance; GEN, gentamicin; AMP, ampicillin.*

### Antibiotic Susceptibility Test

Minimum inhibitory concentrations were determined with the standard agar dilution method on Mueller-Hinton (MH) agar according to the Clinical and Laboratory Standards Institute guidelines (CLSI, 2020). The MICs of β-lactam inhibitors were determined by broth microdilution in cation-adjusted Mueller-Hinton broth (CAMHB). The MIC was interpreted as the β-lactam concentration at which bacterial growth was no longer observed after 20 h of incubation at 37°C. The MICs of ampicillin-avibactam and ampicillin-tazobactam were determined at a constant concentration of 4 mg/L avibactam and tazobactam, and in combination with a series of increasing concentrations of ampicillin, while for amoxicillin-clavulanic acid, a constant concentration ratio (2:1, amoxicillin:clavulanic acid) was applied. MICs for the tested antibiotics were interpreted according to the guidelines of the CLSI (2020); however, for some antibiotics without CLSI interpretation criteria for *P. aeruginosa*, breakpoints for Enterobacteriaceae in the CLSI guidelines were used as a reference. *E. coli* ATCC 25922 was used as a quality control strain. Values are the means of three independent measures.

### Genome Sequencing

A Generay Genomic DNA Miniprep Kit (Shanghai Generay Biotech Co., Ltd., Shanghai, China) was used to extract the total bacterial DNA of *P. wenzhouensis* A20. Genomic DNA was sequenced by both the Illumina HiSeq-2500 and PacBio RS II platforms by Shanghai Personal Biotechnology Co., Ltd. (Shanghai, China). The PacBio long reads were initially assembled by Canu v1.8 (Koren et al., 2017), and hybrid assembly was subsequently conducted using Unicycler v0.4.8 (Wick et al., 2017), with the contigs generated by Canu and all the sequenced reads (including short and long reads) serving as an input. The cyclization of the whole-genome assembly was confirmed through the built-in tools of Unicycler. BWA v0.7.12 (Li and Durbin, 2009) and Genome Analysis Toolkit (McKenna et al., 2010) were used for short read alignment to the draft of the whole-genome assembly to improve assembly quality. Open reading frames (ORFs) were predicted using Prokka v1.14.6 (Seemann, 2014) with default parameters and annotated by the BLAST program with an e-value threshold of 1e-5 against the non-redundant protein sequence (NR) database of the National Center for Biotechnology Information (NCBI) and the UniProt/Swiss-Prot database. Resistance genes were identified using a combination of the ResFinder database (Zankari et al., 2012) and the Comprehensive Antibiotic Resistance Database (CARD) (McArthur et al., 2013). Mobile genetic elements (MGEs) were detected using ISFinder (Siguier et al., 2006) and INTEGRALL (Moura et al., 2009) with default parameters. FastANI v1.31 (Jain et al., 2018) was used to calculate the ANI. ProtParam^1^ was used to predict the molecular weight and pI value of PRC-1. The putative signal peptide cleavage site of PRC-1 was predicted by SignalP 5.0 (Almagro Armenteros et al., 2019). Circular maps of the genome and other relatives were drawn using CGView Server (Petkau et al., 2010). Multiple sequence alignments of PRC-1 and other PDC family β-lactamases were performed using MAFFT v7.475 (Katoh and Standley, 2013). A neighbor-joining phylogenetic tree including PRC-1 and other proteins sharing ≥85% amino acid sequence similarity was reconstructed using MEGAX (Kumar et al., 2018). The resulting tree was visualized using the online tool iTol (Letunic and Bork, 2007). Other bioinformatics tools in this study were applied with Python and Biopython scripts (Cock et al., 2009).

### Cloning of the *bla*_PRC–1_ Gene and Expression and Purification of PRC-1

The nucleotide sequence of the *bla*_PRC–1_ gene with the upstream promoter region was amplified using PrimeSTAR HS DNA Polymerase (Takara Bio Inc., Dalian, China), and *P. wenzhouensis* A20 genomic DNA was used as the template. The primers with restriction endonuclease sites (*Bam*HI and *Hin*dIII for the forward and reverse primers, respectively) are listed in Supplementary Table 1. The PCR product and the cloning vector pUCP24 were digested with both *Bam*HI and *Hin*dIII (Takara Bio Inc.). The resulting DNA fragments were ligated with T4 DNA ligase (Takara Bio Inc.), and the ligated product was transformed into *E. coli* DH5α with the calcium chloride method. The transformants were selected on LB agar plates supplemented with gentamicin (40 μg/mL). Single colonies were inoculated into LB medium supplemented with the same antibiotics and cultured overnight. Plasmids were extracted from the cultures using a Plasmid Mini Extraction Kit (Generay Biotech Co., Ltd.), and the inserts were verified by PCR and further by DNA sequencing (TsingKe, Shanghai, China). The same method was used to clone the ORF of *bla*_PRC–1_ without the signal sequence into the pCold I vector (the primers are listed in Supplementary Table 1), and the recombinant plasmid (pCold I-*bla*_PRC–1_) was transformed into competent *E. coli* BL21 cells. The transformants were selected on LB agar plates supplemented with 100 μg/mL ampicillin. For the expression of PRC-1, the recombinant strain (pCold I-*bla*_PRC–1_/BL21) was cultured overnight in LB medium, diluted 100-fold in fresh medium, and then incubated at 16°C and 250 rpm for 2–3 h, which was followed by the addition of 0.5 mM isopropyl-β-d-thiogalactopyranoside (IPTG, Sigma Chemicals Co., St. Louis, MO, United States) when the OD_600_ reached 0.6 and further incubation for 21 h at 16°C (Choi and Geletu, 2018). His-tagged PRC-1 protein was purified by nickel affinity chromatography with BeyoGold His-tag purification resin (Beyotime, Shanghai, China) and then digested with Enterokinase (GenScript, Nanjing, China) for 40 h at 37°C to remove the His-tag. The protein was identified by SDS-PAGE using a 12% acrylamide separation gel and Coomassie blue G-250 staining.

### Enzyme Kinetic Analysis

Kinetic parameters of hydrolysis for β-lactams by the novel β-lactamase PRC-1 were determined on a UV-VIS spectrophotometer (U-3900, HITACHI, Japan) at 37°C in 10 mM phosphate buffer (pH 7.4) in a final reaction volume of 200 μl. The steady-state kinetic parameters (*k*_*cat*_ and *K*_*m*_) were determined by non-linear regression of the initial reaction rates with the Michaelis-Menten equation in Prism (version 8.0.2) software (GraphPad Software, CA, United States) (Chen et al., 2019). For poor substrates (cefepime, imipenem, and aztreonam), the tested antibiotics were treated as competitive inhibitors of the AmpC enzyme and nitrocefin as a reporter substrate, and the inhibition constants were determined by observing the apparent *K*_*m*_ at various concentrations of inhibitor. Data were analyzed to obtain the values of Vmax and *K*_*m*_ by GraphPad Prism with the competitive model inhibition/non-linear regression curve fit method. *k*_*cat*_ values were determined from the initial rates calculated at saturating substrate concentrations (Faheem et al., 2013). The concentrations of the β-lactamase inhibitors avibactam and clavulanic acid leading to a 50% reduction in hydrolysis of nitrocefin (IC50) were measured after 5 min of preincubation of the enzymes with the inhibitors at 37°C and nitrocefin as the substrate at 100 μM. The IC50 values were determined by non-linear regression analysis (GraphPad Prism, version 8.0.2) using log (inhibitor) vs. response – (three parameters) (Chen et al., 2019). Values are the means of three independent measures.

### Nucleotide Sequence Accession Numbers

The chromosome and *bla*_PRC–1_ gene sequences of *P. wenzhouensis* A20 have been deposited in GenBank under accession numbers CP072610 and MW854031, respectively.

## Results and Discussion

### Genomic Properties of *Pseudomonas wenzhouensis* A20

The strain *P. wenzhouensis* A20 was isolated from sewage discharged from an animal farm in Wenzhou, China. 16S rRNA homologous gene analysis demonstrated that *P. wenzhouensis* A20 showed the closest relationship with the strain *Pseudomonas hydrolytica* DSWY01 (NR_170428.1), at 98.50% identity and 100% coverage. The taxonomy of the genus *Pseudomonas* was developed mainly through phenotypic and biochemical properties (Palleroni, 2010), whereas currently, molecular analysis, as a good supplement, is particularly important. According to previous studies on the phylogeny of *Pseudomonas* based on 16S ribosomal DNA (rDNA) sequences, there was not enough discrimination at the species level (Mulet et al., 2010), and the multilocus sequence analysis (MLSA) approach based on the four housekeeping genes (the 16S rRNA, *gyrB*, *rpoB*, and *rpoD* genes) has subsequently been applied, which provided better results than the former method but could not accurately discriminate the phylogeny of *Pseudomonas* species (Mulet et al., 2010). At present, the new gold standards for species delineation are digital whole-genome comparisons by using genome-to-genome distance calculations (GGDCs) or ANI (Lalucat et al., 2020). According to ANI, the threshold to classify the species of a certain bacterium is a cutoff score of ≥95% between the unclassified bacterium genome and all the bacterial genomes available in the public databases. Given the large number of genomes (∼26,600) from 303 *Pseudomonas* species available in the NCBI database, one genome sequence from each of 40 species which showed the highest similarities of 16S rRNA gene sequences with that of A20 was chosen to perform ANI analysis. Among them, the highest ANI value of 94.25% was found between the A20 genome and that of *Pseudomonas mendocina* EF27 (GCF_008041835.1) (Supplementary Table 3), indicating that strain A20 might represent a novel species of the genus *Pseudomonas*. Moreover, when submitting the genome sequence to GenBank of NCBI, the ANI results calculated by the scientists there also did not identify a species for A20, which further confirmed it to be a novel species.

**FIGURE 1:**
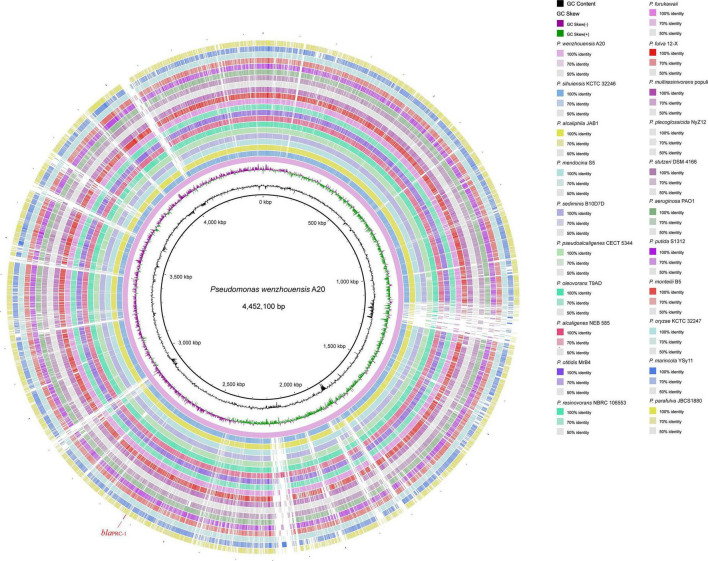
Comparative chromosome map of the *P. wenzhouensis* A20 and 20 other *Pseudomonas* strains using A20 as the reference. Circle 1 (from inside to outside) shows the scale in kb. Circles 2 and 3 show the GC content and GC skew, respectively. Circles 4–25 are the regions homologous to A20 and the other 20 strains, respectively. Similar parts are represented by lines of different shades, and the regions without similar hits leave blank. The strains used for comparison in this study are listed in Supplementary Table 2.

The *P. wenzhouensis* A20 genome consists of a circular chromosome and does not have any plasmid. The chromosome is 4.4 Mb in length with an average GC content of 62.2% and encodes 4,063 ORFs (Table 2). Screening for A20-homologous genomes (>80% nucleotide sequence identity and >30% coverage) in the NCBI nucleotide database showed that most of the close relatives were derived from 20 species. Comparative genomic analysis revealed that the chromosome of *P. wenzhouensis* A20 shared the highest sequence similarities with those of *Pseudomonas sihuiensis* KCTC 32246 chromosome (LT629797.1; 80.83% coverage and 89.54% identity), *Pseudomonas alcaliphila* JAB1 chromosome (CP016162.1; 80.78% coverage and 89.19% identity), and *P. mendocina* S5 (CP013124.1; 79.02% coverage and 88.59% identity). Moreover, A20 is basically similar to other species in conserved backbone sequences (Figure 1), among them, similar sequences are represented by lines of different shades, whereas the blank regions indicate differences among A20 and other species.

**TABLE 2:** General features of the *P. wenzhouensis* A20 genome.

	Chromosome
Size (bp)	4,452,100
GC content (%)	62.20
CDS	4,063
Known proteins	3,601 (88.63%)
Hypothetical proteins	462 (11.37%)
Protein coding (%)	88.12
Average ORF length (bp)	970
Average protein length (aa)	321
tRNAs	83
rRNA operons	(16S-23S-5S) *4

### The Resistance Profile of *Pseudomonas wenzhouensis* A20

Antibiotic susceptibility testing showed that A20 exhibited intermediate resistance to nalidixic acid. *P. wenzhouensis* A20 had the highest MIC levels for fosfomycin (>512 μg/mL), cefazolin (256 μg/mL), and cefoxitin (128 μg/mL) and higher MIC levels for aztreonam (32 μg/mL) and cefotaxime (8 μg/mL) (Table 3). As the breakpoints for fosfomycin, cefazolin, cefoxitin, aztreonam, and cefotaxime were not available for *P. aeruginosa* in CLSI interpretation criteria, the breakpoints for *Enterobacteriaceae* in the CLSI guidelines were referred to, and the MIC values of *P. wenzhouensis* A20 were equivalent to those of resistant enterobacteria for these five antimicrobial agents. The isolate was susceptible to some third- and fourth-generation cephalosporins (e.g., ceftazidime, cefoperazone, cefoselis, and cefepime) and carbapenems (such as meropenem). When analyzing the resistance mechanism of the bacterium, especially for β-lactam antibiotics, we found that only one predicted *ampC* β-lactamase gene was annotated within the whole genome, which showed an identity of more than 50% with the functionally characterized resistance gene *bla*_PDC–211_ (57.4%, MF281075.1). The predicted gene was then cloned, and the resistance function was further determined.

**TABLE 3:** Minimum inhibitory concentrations of antimicrobials for *P. wenzhouensis* A20, the recombinants, and the control strain (μg/mL).

Antibiotic	*P. wenzhouensis* A20	pUCP24-*bla*_PRC–1_/DH5α	pUCP24/DH5α	DH5α	ATCC 25922
Ampicillin	16	16	2	4	2
Cefazolin	256	4	1	1	2
Cefoxitin	128	4	2	4	2
Ceftazidime	1	0.5	0.25	0.25	0.25
Cefepime	0.125	0.125	0.06	0.06	0.125
Cefoperazone	8	0.5	0.5	0.5	0.5
Ceftriaxone	2	0.125	0.03	0.03	0.06
Cefotaxime	8	0.25	0.03	0.03	0.06
Cefoselis	1	0.125	0.06	0.125	0.125
Aztreonam	32	0.125	0.06	0.06	0.25
Meropenem	0.25	0.03	0.015	0.015	0.015
Amoxicillin	8	32	1	2	8
Amoxicillin-clavulanic acid	8	32	1	1	4
Ampicillin-avibactam	8	4	1	1	2
Ampicillin-tazobactam	16	16	2	2	2
Fosfomycin	>512	–	–	–	2
Nalidixic acid	16	–	–	–	2

### Functional Characterization of the PRC-1 β-Lactamase

To determine the resistance characteristics of *bla*_PRC–1_ to β-lactam antibiotics, the coding sequence of *bla*_PRC–1_ together with its upstream promoter region was amplified and cloned into the pUCP24 vector and then transformed into *E. coli* DH5α. The results revealed that *bla*_PRC–1_ conferred resistance to some penicillins and first- and third-generation cephalosporins (Table 3). Compared with the control strains (DH5α and pUCP24/DH5α), the recombinant strain (pUCP24-*bla*_PRC–1_/DH5α) exhibited increased MIC levels for ampicillin, cefotaxime, and amoxicillin by 8-, 8-, and 32-fold, respectively. The MICs of ceftriaxone, cefazolin, penicillin G, and cefotaxime exhibited a lower increase of 4-fold. However, the recombinant strain did not show any MIC level changes for carbapenems or monobactams. The activity of PRC-1 was poorly inhibited by classical class A β-lactamase inhibitors such as clavulanic acid and tazobactam, while a significant decrease in resistance to ampicillin was observed in the presence of avibactam.

**FIGURE 2:**
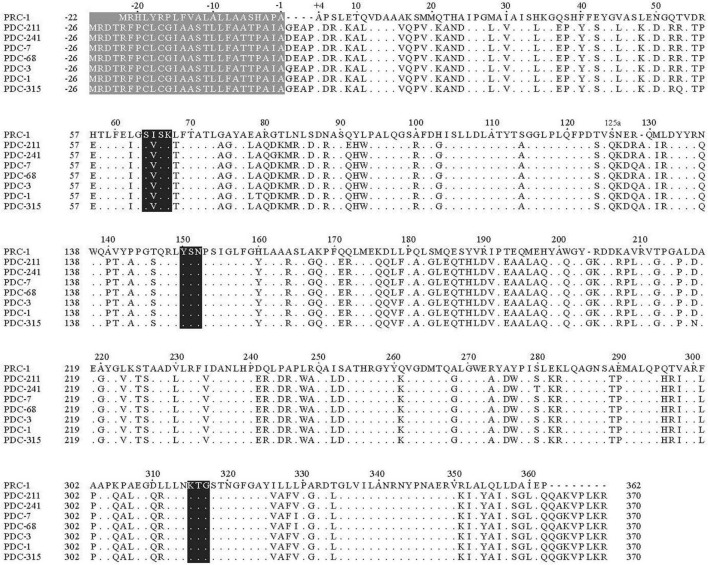
Multiple alignment of the deduced amino acid sequences of PRC-1 and other chromosomal class C β-lactamases. Three conserved motifs are shaded in black. Identical amino acids are indicated by dots, and the absence of amino acids at those positions is shown with hyphens. The numbering of PRC-1 is based on a standard numbering scheme for class C β-lactamases (Mack et al., 2020). The sequences and their accession numbers are PDC-211 (ARX71249), PDC-241 (AUT06978), PDC-7 (ACQ82812), PDC-68 (AIG20005), PDC-3 (ACQ82808), PDC-1 (AAM08945), and PDC-315 (AYF58375).

Multiple sequence alignment of the deduced amino acid sequence of PRC-1 with those of functionally characterized β-lactamases revealed that PRC-1 had identities of 57.7, 57.5, 57.3, 57.1, 57.0, 56.8, and 56.8% with PDC-211, PDC-241, PDC-7, PDC-68, PDC-3, PDC-1, and PDC-315, respectively. The deduced amino acid sequence carries the characteristic catalytic residues of the serine active site of β-lactamases, including motifs of S-X-X-K (serine-isoleucine-serine-lysine) with the initial amino acid sequence number at positions 64–67, Y-S-N (tryptophan-serine-asparagine) at positions 150–152, and K-T-G (lysine-threonine-glycine) at positions 315–317 (Figure 2).

Because no detailed resistance spectrum is available for *bla*_PDC–211_, *bla*_PDC–241_, *bla*_PDC–7_, and *bla*_PDC–68_, which shared relatively higher amino acid sequence identities with PRC-1, we compared the resistance profile of *bla*_PDC–3_ with that of *bla*_PRC–1_. PDC-3 (ACQ82808) is a chromosomal AmpC β-lactamase of *P. aeruginosa* with an amino acid identity of 57.0% (216/379) with PRC-1. Unlike *bla*_PDC–3_, *bla*_PRC–1_ exhibited a relatively narrow resistance spectrum and did not confer resistance to piperacillin, cefepime, ceftazidime, or even aztreonam. In addition, *bla*_PRC–1_ showed lower MIC values than *bla*_PDC–3_ against other β-lactam antibiotics (Barnes et al., 2018). Additionally, we found that the *bla*_PRC–1_ gene did not show any resistance to cefoxitin but showed a relatively lower MIC level for cefazolin, even though *P. wenzhouensis* A20 showed much higher MIC levels for them. This finding may indicate the existence of unknown resistance mechanisms within *P. wenzhouensis* A20.

The novel β-lactamase gene *bla*_PRC–1_ is 1140 bp in size and encodes a 379-amino acid putative protein. The mature protein has a predicted molecular weight of 41.48 kDa and a predicted pI of 6.44. The secretory precursor peptide, which consists of 22 amino acids, in PRC-1 is defined by alanine residues at positions 22 and 23. The molecular weight of the protein without the signal peptide is 39.07 kDa. The purified protein PRC-1 exhibited a single band on SDS-PAGE, and its molecular size was in agreement with the predicted one. The kinetic parameters of PRC-1 were determined by measuring the rates of catalysis for various β-lactam antibiotics at different substrate concentrations. The results demonstrated that PRC-1 was a typical cephalosporinase with a high *k*_*cat*_ and strong hydrolytic activity (*k*_*cat*_/*K*_*m*_ ratios were 220 × 10^–2^ μM^–1⋅^s^–1^) for the first-generation cephalosporin cefazolin. PRC-1 showed moderate hydrolysis activities against some third-generation cephalosporins (cefotaxime and ceftriaxone) and penicillins (ampicillin and benzylpenicillin) but very poor hydrolytic activity against ceftazidime (the third-generation cephalosporin) (Table 4). However, the result of the enzyme kinetic hydrolytic activity test was not completely consistent with the MIC level change of the recombinant strain (pUCP24-*bla*_PRC–1_/DH5α) in the antimicrobial susceptibility test (Table 3). For example, PRC-1 showed hydrolytic activity against ceftazidime, but the recombinant strain carrying *bla*_PRC–1_ did not exhibit a significant change in the MIC of ceftazidime compared with that for the control bacteria. This result may be attributed to its low activity *in vitro*. A similar case in a previous study reported that BAT-2 and BSU-2 exhibited slight hydrolytic activities against ampicillin, but the two genes did not show detectable resistance activities to ampicillin in the recombinant strains carrying them (Toth et al., 2016). We also found that the catalytic efficiency (*k*_*cat*_/*K*_*m*_) of ceftazidime was the lowest among all the tested substrates. Moreover, the affinity of poor substrates, such as aztreonam, cefepime, or imipenem, was higher than those of other substrates in the competitive inhibition test with nitrocefin; the *K*i values for aztreonam, cefepime, and imipenem were (307 ± 21) × 10^–3^, (134 ± 20) × 10^–3^, and (72 ± 7) × 10^–3^ μM, respectively, and no hydrolysis was determined for these substrates at saturating concentrations (Table 4), which was in line with the susceptibility test results. Conversely, PDC-3, which shares 57.0% global amino acid identity with PRC-1, exhibited hydrolytic activities against cefepime and imipenem (Rodriguez-Martinez et al., 2009). Both β-lactamases showed similar catalytic efficiencies for benzylpenicillin, whereas PRC-1 displayed higher *K*_*m*_ and *k*_*cat*_ values, revealing that higher turnover numbers are compensated by higher *K*_*m*_ values (Table 4). The IC50 (50% inhibitory concentration) of β-lactamase inhibitors showed that avibactam (IC50: 0.0003922 μM) has a strong inhibitory effect on PRC-1, while clavulanic acid (IC50: 23.43 μM) has a weaker inhibitory effect. This result is in line with the properties of β-lactam inhibitors for AmpC enzymes.

**TABLE 4:** Kinetic parameters of PRC-1 for β-lactam antibiotics.

Substrate	*K*_*m*_ or *K*_*i*_ (μM)*^a^*	*k*_*cat*_ (s^–1^)*^a^*	*k*_*cat*_/*K*_*m*_ (μM^–1⋅^s^–1^)
Benzylpenicillin	126 ± 18	123 ± 17	98 × 10^–2^
Ampicillin	65 ± 7	23 ± 2	35 × 10^–2^
Cefazolin	210 ± 31	463 ± 28	220 × 10^–2^
Cefotaxime	529 ± 81	156 ± 10	29 × 10^–2^
Ceftriaxone	68 ± 14	32 ± 4	47 × 10^–2^
Ceftazidime	253 ± 20	(249 ± 31) × 10^–2^	1 × 10^–2^
Aztreonam	(307 ± 21) × 10^–3^	NH*^b^*	–
Imipenem	(134 ± 20) × 10^–3^	NH*^b^*	–
Cefepime	(72 ± 7) × 10^–3^	NH*^b^*	–

*^a^ Values are means ± standard deviations.*

*^b^ NH, no detectable hydrolysis.*

### Taxonomic Distribution of *bla*_PRC–1_

**FIGURE 3:**
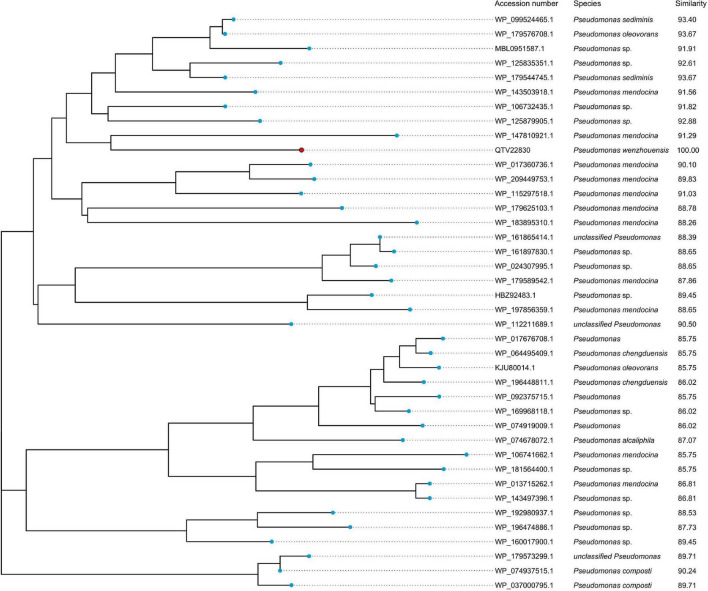
Phylogenetic analysis of PRC-1 with other putative class C β-lactamases (≥85% amino acid similarity). PRC-1 from this study is represented with a red dot.

To analyze the possible origin of *bla*_PRC–1_, a total of 40 predicted proteins with an amino acid similarity ≥85% were retrieved from the NCBI-nr database, and all of them were from the genus *Pseudomonas*. The phylogenetic tree showed that PRC-1 was closest to a putative AmpC β-lactamase found in *P. mendocina* (WP_147810921.1), and they shared the highest amino acid similarity (91.29% identity and 100% coverage) (Figure 3). These findings indicate the importance of *Pseudomonas* as a reservoir for PRC-1-like relatives, and additional *Pseudomonas* genomes must be sequenced to find proteins with higher identities with PRC-1.

### Analysis of Genetic Environment of bla_PRC–1_

**FIGURE 4:**
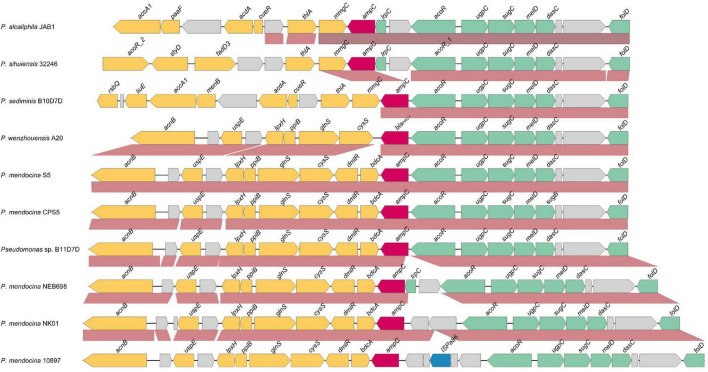
Comparative genomics analysis of the genetic context of *bla*_PRC–1_ with the sequences carrying their homologous genes. The direction of genes is shown *via* an arrow. The *bla*_PRC–1_ gene and putative *ampC* (*bla*_PRC–1_-like) genes are colored in red, and the other genes are colored based on gene function classification. The predicted hypothetical genes are in gray. The sequences and their accession numbers are as follows: *P. alcaliphila* JAB1 chromosome (CP016162.1), *P. sihuiensis* 32246 chromosome (LT629797.1), *P. sediminis* B10D7D chromosome (CP060009.1), *P. mendocina* S5.2 chromosome (CP013124.1), *P. mendocina* CPS5 chromosome (CP060288.1), *Pseudomonas* sp. B11D7D chromosome (CP060008.1), *P. mendocina* NEB698 chromosome (CP027657.1), *P. mendocina* NK-01 chromosome (CP002620.1), and *P. mendocina* NCTC 10897 chromosome (LR134290.1).

To analyze the genetic environments of *bla*_PRC–1_ and its relatives, nine sequences of approximately 20 kb in length with *bla*_PRC–1_-like genes at the center were retrieved from the NCBI nucleotide database, and these *bla*_PRC–1_-like genes shared over 85% amino acid similarities with PRC-1. No mobile genetic element was predicted in its surrounding area. Comparative genomic analysis of the 10 sequences (including the one from this work, *P*. *wenzhouensis* A20) revealed that the upstream regions (from *folD* to *acoR*) of the *bla*_PRC–1_ and *bla*_PRC–1_-like (*ampC*) genes of all 10 sequences have a conserved structure in terms of gene context and gene order. Three sequences (*P. mendocina* S5, *P. mendocina* 5, and *Pseudomonas* sp. B11D7D) have the most similar structure to the sequence from this study, except two additional genes (*dmlR* and *bdcA*) downstream of the *bla*_PRC–1_-like (*ampC*) genes that were found in these three sequences. Three sequences (*P. mendocina* NEB698, *P. mendocina* NK-01, and *P. mendocina strain* NCTC 10897) had an extra two or more genes upstream of the *bla*_PRC–1_-like (*ampC*) genes. The remaining three sequences had the same (*P. sediminis* B10D7D) or nearly the same (*P. alcaliphila* JAB1 and *P*. *sihuiensis* 32246) gene context as that of the one from this work in the regions downstream of *bla*_PRC–1_; however, the upstream regions of these three sequences were totally different from *P*. *wenzhouensis* A20. All 10 sequences were from the genus *Pseudomonas*, and the three sequences with the most similarity with *P*. *wenzhouensis* A20 were from *P. mendocina* (*P. mendocina* S5 and *P. mendocina* CPS5) and an unclassified *Pseudomonas* (*P*. sp. B11D7D) (Figure 4). These results suggested that the gene context of *bla*_PRC–1_ and its relatives is conserved in species of the genus *Pseudomonas*.

## Conclusion

In this work, we characterized a novel AmpC β-lactamase-encoding gene, *bla*_PRC–1_, in the chromosome of *P. wenzhouensis* A20, an isolate of a newly classified species of the *Pseudomonas* genus. *bla*_PRC–1_ shared the highest amino acid similarity (57.7%) with the functionally characterized AmpC enzyme PDC-211 from *P. aeruginosa* and conferred resistance to β-lactam antibiotics, including some cephalosporins, such as cefazolin, ceftriaxone, and cefotaxime. Similar to other AmpC β-lactamases, the novel β-lactamase is strongly inhibited by avibactam, but inhibitors of class A enzymes such as clavulanic acid have a weaker inhibitory effect against it. *bla*_PRC–1_-like genes with amino acid similarities of more than 85% were identified in many bacteria of different species, and further research would be carried out to determine the functions of these potential resistance genes. Deciphering more antibacterial resistance mechanisms will be crucial to assist clinics in using effective antibiotics to treat infections caused by unusual pathogens.

## Data Availability Statement

The datasets presented in this study can be found in online repositories. The names of the repository/repositories and accession number(s) can be found in the article/Supplementary Material.

## Author Contributions

HZ, MZ, and QB: conception and design of the study. JLL, XL, WS, MG, and PR: acquisition of data. PZ, XD, KZ, JLL, CF, XL, and KL: data analysis and interpretation. PZ, XD, KZ, JWL, QB, and HZ: drafting of manuscript. PZ, XD, WS, MG, QL, and XZ: performed the experiments. All authors contributed to the article and approved the submitted version.

## Conflict of Interest

The authors declare that the research was conducted in the absence of any commercial or financial relationships that could be construed as a potential conflict of interest.

## Publisher’s Note

All claims expressed in this article are solely those of the authors and do not necessarily represent those of their affiliated organizations, or those of the publisher, the editors and the reviewers. Any product that may be evaluated in this article, or claim that may be made by its manufacturer, is not guaranteed or endorsed by the publisher.

## Footnotes

1
https://web.expasy.org/protparam/
