# Supplementary material for: Characterization of a Novel Chromosome-Encoded AmpC β-Lactamase Gene, blaPRC–1, in an Isolate of a Newly Classified Pseudomonas Species, Pseudomonas wenzhouensis A20, From Animal Farm Sewage
Source: Front Microbiol. 2021 Dec 17;12:732932. doi: 10.3389/fmicb.2021.732932 (PMC8719060; doi:10.3389/fmicb.2021.732932)
Supplement: Supplementary file 1 [file Table_1.DOCX]

**TABLE S1** | Primers used in this study.

| Primer *^a^* | Sequence (5’–3’) *^b^* | Restriction  endonuclease | Vector | Amplicon size  (bp) | Annealing  temperature (°C) |
| --- | --- | --- | --- | --- | --- |
| *pro*-*bla*_PRC-1_-F | CGGGATCCGTACTGGAGCTGTTCCAGCAT | *Bam*HI | pUCP24 | 1,547 | 59 |
| *pro*-*bla*_PRC-1_-R | CCAAGCTTCTACGGCTCGATCGCGTC | *Hin*dIII |  |  |  |
| *orf*-*bla*_PRC-1_-F | CGCGGATCCGACGACGACGACAAGGCACCGCAAACGGAAAAACAGG | *Bam*HI+DDDDK | pCold I | 1,140 | 60 |
| *orf*-*bla*_PRC-1_-R | CCAAGCTTCTACGGCTCGATCGCGTC | *Hin*dIII |  |  |  |

^a^ Primers with “orf” were used to clone the ORF of the *bla*_PRC-1_ gene, and primers with “pro” were used to clone the *bla*_PRC-1_ gene with its promoter region.

^b^ The underlined sequences represent the restriction endonuclease sites and their protective bases.
