# Supplementary material for: Characterization of a Novel Chromosome-Encoded AmpC β-Lactamase Gene, blaPRC–1, in an Isolate of a Newly Classified Pseudomonas Species, Pseudomonas wenzhouensis A20, From Animal Farm Sewage
Source: Front Microbiol. 2021 Dec 17;12:732932. doi: 10.3389/fmicb.2021.732932 (PMC8719060; doi:10.3389/fmicb.2021.732932)
Supplement: Supplementary file 2 [file Table_2.docx]

**TABLE S2** | The strains used for comparative genomic analysis in this study.

| **Species** | **Strain** | **Accession number** |
| --- | --- | --- |
| *P. aeruginosa* | PAO1 | NC_002516.2 |
| *P. alcaligenes* | NEB 585 | CP014784.1 |
| *P. alcaliphila* | JAB1 | CP016162.1 |
| *P. fulva* | 12-X | CP002727.1 |
| *P. furukawaii* | - | AP014862.1 |
| *P. marincola* | YSy11 | LR215729.1 |
| *P. mendocina* | S5 | CP013124.1 |
| *P. monteilii* | B5 | CP022562.1 |
| *P. multiresinivorans* | populi | CP048833.1 |
| *P. oleovorans* | T9AD | LR130779.2 |
| *P. oryzae* | KCTC 32247 | LT629751.1 |
| *P. otitidis* | MrB4 | AP022642.1 |
| *P. parafulva* | JBCS1880 | CP031641.1 |
| *P. plecoglossicida* | NyZ12 | CP010359.1 |
| *P. pseudoalcaligenes* | CECT 5344 | HG916826.1 |
| *P. putida* | S13.1.2 | CP010979.1 |
| *P. resinovorans* | NBRC 106553 | AP013068.1 |
| *P. sediminis* | B10D7D | CP060009.1 |
| *P. sihuiensis* | KCTC 32246 | LT629797.1 |
| *P. stutzeri* | DSM 4166 | CP002622.1 |
