# Supplementary material for: Characterization of a Novel Chromosome-Encoded AmpC β-Lactamase Gene, blaPRC–1, in an Isolate of a Newly Classified Pseudomonas Species, Pseudomonas wenzhouensis A20, From Animal Farm Sewage
Source: Front Microbiol. 2021 Dec 17;12:732932. doi: 10.3389/fmicb.2021.732932 (PMC8719060; doi:10.3389/fmicb.2021.732932)
Supplement: Supplementary file 3 [file Table_3.docx]

**TABLE S3 |** The strains used for ANI analysis in this study.

| Species* | ANI (%) | Ref_genome |
| --- | --- | --- |
| *P. amygdali* | 79.0424 | GCF_000145945.2 |
| *P. savastanoi* | 79.082 | GCF_003702785.1 |
| *P. syringae* | 79.3276 | GCF_001400955.1 |
| *P. taiwanensis* | 79.5574 | GCF_000425785.1 |
| *P. plecoglossicida* | 80.1974 | GCF_003391255.1 |
| *P. guineae* | 80.2603 | GCF_900113745.1 |
| *P. balearica* | 80.6614 | GCF_016622365.1 |
| *P. songnenensis* | 80.8687 | GCF_003696315.1 |
| *P. kunmingensis* | 80.9917 | GCF_013409135.1 |
| *P. chloritidismutans* | 81.0484 | GCF_000495915.1 |
| *P. chlororaphis* | 81.0664 | GCF_000963835.1 |
| *P. xanthomarina* | 81.0954 | GCF_900108535.1 |
| *P. fluorescens* | 81.0958 | GCF_900636635.1 |
| *P. aeruginosa* | 81.2211 | GCF_000006765.1 |
| *P. resinovorans* | 81.2263 | GCF_000423545.1 |
| *P. mangrovi* | 81.3259 | GCF_003052585.1 |
| *P. pharmacofabricae* | 81.4376 | GCF_002835605.1 |
| *P. indica* | 81.533 | GCF_002091635.1 |
| *P. stutzeri* | 81.589 | GCF_002890915.1 |
| *P. flavescens* | 81.7289 | GCF_002091575.1 |
| *P. peli* | 81.7407 | GCF_900099645.1 |
| *P. knackmussii* | 81.8261 | GCF_009911755.1 |
| *P. anguilliseptica* | 81.9637 | GCF_900105355.1 |
| *P. putida* | 82.0725 | GCF_000412675.1 |
| *P. otitidis* | 82.202 | GCF_011397855.1 |
| *P. argentinensis* | 82.2812 | GCF_900113905.1 |
| *P. straminea* | 82.312 | GCF_900112645.1 |
| *P. borbori* | 82.6363 | GCF_900115555.1 |
| *P. alcaligenes* | 83.2 | GCF_000467105.1 |
| *P. khazarica* | 87.2095 | GCF_017915135.1 |
| *P. composti* | 88.4498 | GCF_013407905.1 |
| *P. sediminis* | 88.9478 | GCF_013409125.2 |
| *P. hydrolytica* | 89.0111 | GCF_004123735.1 |
| *P. toyotomiensis* | 89.6142 | GCF_000974625.1 |
| *P. alcaliphila* | 89.7479 | GCF_001941865.1 |
| *P. chengduensis* | 89.8589 | GCF_900102635.1 |
| *P. indoloxydans* | 90.9257 | GCF_003052605.1 |
| *P. indoloxydans* | 90.9257 | GCF_003052605.1 |
| *P. oleovorans* | 91.244 | GCF_900109155.1 |
| *P. mendocina* | 94.2482 | GCF_008041835.1 |

*The genome sequences of the strains sharing >95% 16S RNA gene similarities with A20 were chosen for the ANI analysis.
